# Supplementary material for: Ecology of inorganic sulfur auxiliary metabolism in widespread bacteriophages
Source: Nat Commun. 2021 Jun 9;12:3503. doi: 10.1038/s41467-021-23698-5 (PMC8190135; doi:10.1038/s41467-021-23698-5)
Supplement: Supplementary file 10 — Description of Additional Supplementary Files [file 41467_2021_23698_MOESM10_ESM.docx]

**Description of additional supplementary information**

**Title: Supplementary Data 1.**

**Description;** Details of mVCs used in this study. Metadata was recovered from IMG/VR.

**Title: Supplementary Data 2.**

**Description**: Protein annotations generated using VIBRANT for each mVC.

**Title: Supplementary Data 3.**

**Description**: The phage and bacterial *dsrA* gene abundance percentage and phage *dsrA* gene expression table.

**Title: Supplementary Data 4.**

**Description**: The phage and bacterial *soxYZ* gene abundance percentage.

**Title: Supplementary Data 5.**

**Description**: Validation of mVC sequences as true phage identifications.

**Title: Supplementary Data 6.**

**Description**: Phage AMG pairs and dN/dS calculations. This table is corresponding to Supplementary Figure 6. The table header corresponds to the output generated from dnds_from_drep.py.

**Title: Supplementary Data 7.**

**Description**: Statistics for validation of the protein grouping method. 100 iterations of 94 random Caudovirales phages from NCBI RefSeq to 94 mVCs are compared.
