# Supplementary material for: Ecology of inorganic sulfur auxiliary metabolism in widespread bacteriophages
Source: Nat Commun. 2021 Jun 9;12:3503. doi: 10.1038/s41467-021-23698-5 (PMC8190135; doi:10.1038/s41467-021-23698-5)
Supplement: Supplementary file 11 — Reporting Summary [file 41467_2021_23698_MOESM11_ESM.pdf]

nature research

Corresponding author(s): Karthik Anantharaman

Last updated by author(s): May 5, 2021

Reporting Summary

Nature Research wishes to improve the reproducibility of the work that we publish. This form provides structure for consistency and transparency in reporting. For further information on Nature Research policies, see our Editorial Policies and the Editorial Policy Checklist.

Statistics

For all statistical analyses, confirm that the following items are present in the figure legend, table legend, main text, or Methods section.

n/a	Confirmed	
☒	☐	The exact sample size (*n*) for each experimental group/condition, given as a discrete number and unit of measurement
☒	☐	A statement on whether measurements were taken from distinct samples or whether the same sample was measured repeatedly
☒	☐	The statistical test(s) used AND whether they are one- or two-sided<br>*Only common tests should be described solely by name; describe more complex techniques in the Methods section.*
☒	☐	A description of all covariates tested
☒	☐	A description of any assumptions or corrections, such as tests of normality and adjustment for multiple comparisons
☒	☐	A full description of the statistical parameters including central tendency (e.g. means) or other basic estimates (e.g. regression coefficient) AND variation (e.g. standard deviation) or associated estimates of uncertainty (e.g. confidence intervals)
☒	☐	For null hypothesis testing, the test statistic (e.g. F, t, r) with confidence intervals, effect sizes, degrees of freedom and P value noted<br>*Give P values as exact values whenever suitable.*
☒	☐	For Bayesian analysis, information on the choice of priors and Markov chain Monte Carlo settings
☒	☐	For hierarchical and complex designs, identification of the appropriate level for tests and full reporting of outcomes
☒	☐	Estimates of effect sizes (e.g. Cohen's *d*, Pearson's *r*), indicating how they were calculated

Our web collection on statistics for biologists contains articles on many of the points above.

Software and code

Policy information about availability of computer code

Data collection	IMG/VR v2
Data analysis	Prodigal v2.6.3; VIBRANT v1.2.1, VirSorter v1.0.3; mash v2.0; nucmer v3.1; DIAMOND BLAST v0.9.14.115; vConTACT2 v0.9.5; Cytoscape v3.7.2; Matplotlib v3.0.0; Basemap v1.20; MAFFT v7.388; Geneious Prime 2019.0.3; dRep v2.6.2; dnds_from_drep.py (https://github.com/MrOlm/bacterialEvolutionMetrics); Seaborn v0.8.1; EasyFig v2.2.2; CD-HIT v4.7; IQ-TREE v1.6.9; iTOL online server; Sickle v1.33; Bowtie2 v2.3.4.1; metaWRAP v1.0.2; SortMeRNA v2.0; R package "ComplexHeatmap"; Blastn v2.2.31; tBlastx v2.2.31; CRT v1.2; custom scripts for this study can be found at github.com/AnantharamanLab/Kieft_and_Zhou_et_al._2020

For manuscripts utilizing custom algorithms or software that are central to the research but not yet described in published literature, software must be made available to editors and reviewers. We strongly encourage code deposition in a community repository (e.g. GitHub). See the Nature Research guidelines for submitting code & software for further information.

Data

Policy information about availability of data

All manuscripts must include a data availability statement. This statement should provide the following information, where applicable:

- Accession codes, unique identifiers, or web links for publicly available datasets
- A list of figures that have associated raw data
- A description of any restrictions on data availability

All IMG/VR sequences are available at https://img.jgi.doe.gov/cgi-bin/vr/main.cgi and https://genome.jgi.doe.gov/portal/pages/dynamicOrganismDownload.jsf?organism=IMG_VR. Sequences from identified vMAGs are available publicly and described in Supplementary Data 1 and 2. Any other relevant data are available from the authors upon request.

Field-specific reporting

Please select the one below that is the best fit for your research. If you are not sure, read the appropriate sections before making your selection.

☐ Life sciences ☐ Behavioural & social sciences ☒ Ecological, evolutionary & environmental sciences

For a reference copy of the document with all sections, see nature.com/documents/nr-reporting-summary-flat.pdf

Ecological, evolutionary & environmental sciences study design

All studies must disclose on these points even when the disclosure is negative.

Study description	This study included the identification of phages encoding auxiliary metabolic genes for dissimilatory sulfur metabolism. Phages were identified according to annotations on the IMG/VR website (see Research Sample). The identified phages were screened to validate them as phages rather than microbial contamination. The phage shared gene content, taxonomy, distribution and abundances (phages, genes and transcripts) were compared.
Research sample	The phages were identified from a publicly available metagenomic-centric database (IMG/VR v2, https://img.jgi.doe.gov/cgi-bin/vr/main.cgi). Other publicly available metagenomic reads and metatranscriptomic reads were also used (e.g., Tara Oceans). All source data is publicly available.
Sampling strategy	The entire IMG/VR v2 database was queried for relevant phages. The sample size (i.e., all sequences present on IMG/VR v2) was the maximum available size and therefore sufficient for analyses.
Data collection	Data was originally collected by Simon Roux by querying the IMG/VR database for genes related to dissimilatory sulfur metabolism. The resulting hits were downloaded, processed and analyzed by Kristopher Kieft and Zhichao Zhou. All relevant methods of phage DNA extraction and sequencing was not performed in this study and can be found on the IMG/VR website under each respective phage project number (provided in manuscript supplemental material).
Timing and spatial scale	The phage genomes used for this study were acquired in October 2018 based on a single query to IMG/VR v2. All relevant phage data was collected during this single query and processed later.
Data exclusions	No data was excluded.
Reproducibility	All raw data (i.e., phage scaffolds and metagenomic/metatranscriptomic reads) are publicly available for download. Complete workflows of custom analysis scripts are provided with documentation on the GitHub page provided in Code Availability. All software used is provided with version and usage information. Using the provided phage scaffolds, reads, analysis scripts and software versions, all analyses can be reproduced as shown in the manuscript.
Randomization	Randomization is not applicable to the analysis of phage genomes from metagenomic samples. All phage genomes on IMG/VR v2 were explicitly searched for sulfur genes of interest, and all analyses were performed on the direct outputs of this search. Therefore, randomization was not used, nor was it applicable for successful non-biased analyses.
Blinding	Blinding was not applicable to this study. Knowledge of the phage genomes, genes and features was required for analysis. The exclusion of blinding to the authors' knowledge did not impact the quality or accuracy of the results.

Did the study involve field work? ☐ Yes ☒ No

Reporting for specific materials, systems and methods

We require information from authors about some types of materials, experimental systems and methods used in many studies. Here, indicate whether each material, system or method listed is relevant to your study. If you are not sure if a list item applies to your research, read the appropriate section before selecting a response.

Materials & experimental systems

n/a	Involved in the study	
☒	☐	Antibodies
☒	☐	Eukaryotic cell lines
☒	☐	Palaeontology and archaeology
☒	☐	Animals and other organisms
☒	☐	Human research participants
☒	☐	Clinical data
☒	☐	Dual use research of concern

Methods

n/a	Involved in the study	
☒	☐	ChIP-seq
☒	☐	Flow cytometry
☒	☐	MRI-based neuroimaging
